# Long non-coding RNA *H19* deficiency ameliorates bleomycin-induced pulmonary inflammation and fibrosis

**DOI:** 10.1186/s12931-020-01534-6

**Published:** 2020-11-02

**Authors:** Xiaoyu Wan, Xinbei Tian, Jun Du, Ying Lu, Yongtao Xiao

**Affiliations:** 1 Department of Respiratory Medicine, Shanghai Pulmonary Hospital, Tongji University School of Medicine, Shanghai, China; 2 Shanghai Institute for Pediatric Research, Shanghai, China; 3 Department of Pediatric Surgery, Xin Hua Hospital, School of Medicine, Shanghai Jiao Tong University, No. 1665, Kong Jiang Road, Shanghai, China

**Keywords:** Pulmonary fibrosis, lncRNA *H19*, Smad, S1pr2

## Abstract

**Background:**

The poor understanding of pathogenesis in idiopathic pulmonary fibrosis (IPF) impaired development of effective therapeutic strategies. The aim of the current study is to investigate the roles of long non-coding RNA *H19* (lncRNA *H19*) in the pulmonary inflammation and fibrosis of IPF.

**Methods:**

Bleomycin was used to induce pulmonary inflammation and fibrosis in mice. The mRNAs and proteins expression in lung tissues was determined by quantitative real-time polymerase chain reaction (qRT-PCR) and western blot. *H19* knockout (*H19*^−/−^) mice were generated by CRISPR/Cas9.

**Results:**

The expression of *H19* mRNA was up-regulated in fibrotic lungs patients with IPF as well as in lungs tissues that obtained from bleomycin-treated mice. *H19*^−/−^ mice suppressed bleomycin-mediated pulmonary inflammation and inhibited the Il6/Stat3 signaling. *H19* deficiency ameliorated bleomycin-induced pulmonary fibrosis and repressed the activation of TGF-β/Smad and S1pr2/Sphk2 in the lungs of bleomycin-treated mice.

**Conclusions:**

Our data suggests that *H19* is a profibrotic lncRNA and a potential therapeutic target for IPF.

## Background

Idiopathic pulmonary fibrosis (IPF) is a progressive and highly lethal pulmonary fibrotic lung disease with poor treatment and unknown etiology, which rises significantly with age and higher in men [1–4]. Patients with IPF present similar characteristics to the usual interstitial pneumonia (UIP), including extracellular matrix deposition, alveolar architectural disruption, and subpleural honeycombing [5]. The patients with IPF usually have clinical experiences from cough to respiratory insufficiency and have a median survival time of 3 to 5 years after diagnosis [1, 4]. Unfortunately, there are currently no effective therapies capable of stabilizing or improving lung function for patients with IPF.

Long non-coding RNAs (lncRNAs) are defined as non-protein encoding RNA molecules that are more than 200 bp long in length [6]. lncRNAs have been shown to play important roles in different physiological activities, such as gene imprinting, cell proliferation, differentiation, apoptosis, migration, and immune responses [7, 8]. Recent studies have shown that aberrant expression of lncRNAs are associated with a number of human diseases, including cardiovascular, neurodegenerative, lung diseases, tumors and infections [9–15]. The lncRNA *H19* is an imprinted and maternally expressed gene that plays a vital role in the controlling the cell proliferation and differentiation [16–18]. The others and our recent studies both indicate that hepatic *H19* level is correlated with the severity of cholestatic injury and liver fibrosis in mice [19, 20]. Furthermore, *H19* was also related to progression of lung cancer and lung fibrosis [21–24]. Although these studies suggest a causal link among *H19* and pulmonary injury, it remains unknown whether and to what extent *H19* is involved in the regulation of pulmonary fibrosis in vivo. In present study, we identified *H19* as an up-regulated lncRNA in the lungs of pulmonary fibrosis. We further determined the functional roles and underlying mechanisms of *H19* in pulmonary fibrosis, which suggested *H19* acts as a profibrotic lncRNA in the lungs.

## Materials and methods

### Materials

Hydroxyproline Assay Kit (Cat. No. MAK008-1KT, Sigma-Aldrich, St. Louis, MO), PowerUp SYBR-Green Master Mix kit (Cat. No. A25742) and a High Capacity cDNA Reverse Transcription kit (Cat. No. 4368814) were obtained from Applied Biosystems (Foster City, CA). NuPAGE 10% Bis–Tris gel (Cat. No. NP0301BOX, Invitrogen, Carlsbad, CA). Bleomycin (Cat. No. HY-17565, MedChemExpress, LLC, NJ), The antibodies used in this study were showed in Additional file 1: Table S1.

### A mouse model of bleomycin-induced pulmonary fibrosis

*H19*^*−/−*^ (*H19* ΔExon1-5) mice were generated by CRISPR/Cas9-mediated genome engineering in C57BL/6J mice as our previously described [20]. The animal procedures were approved by the Shanghai Jiao tong University School of Medicine affiliated Xin Hua hospital Animal Care and Use Committee (XHEC-F-2020-008). The mice (about 8 weeks old) were divided into four groups: wild type (*Wt*) sham (n = 8–12), *H19*^*−/−*^ sham (n = 6–10), wild type (*Wt*) treated with bleomycin (BLM) (n = 8–12) and *H19*^*−/−*^ treated with bleomycin (BLM) (n = 8–12). For bleomycin administration, mice were anaesthetized with 2% isoflurane, and then instilled intratracheally with bleomycin (3.5 mg/kg body weight) in 100 μl PBS, as previously described [25, 26]. After 4 weeks, the lung tissues were collected for RNA, protein, collagen content analyses and histological analysis. The degree of fibrosis was quantitated using an Ashcroft score in a blinded manner according to the described method [27].

### Fluorescence in situ hybridization (FISH)

H19 FISH in mouse lung tissue was performed using a commercially available RNAscope Multiplex Fluorescent Reagent Kit v2 (Advanced Cell Diagnostics, Newark, CA) and RNAscope^®^ Probe-Mm-H19 (#423751, Advanced Cell Diagnostics, Newark, CA) according to the manufacturer’s instruction. Fluorescent staining targeting Sftpc protein was performed followed H19 FISH.

### Quantitative real-time polymerase chain reaction (qRT-PCR)

Total RNA was extracted from left lung of mice using the RNeasy kit (Qiagen, Hilden, Germany) according to the protocol of the manufacture and 2 μg of total RNA was used to synthesize the 1st cDNA using a High Capacity cDNA Reverse Transcription kit (Applied Biosystems, Foster City, CA). The real-time PCR reactions were performed on the ViiA 7 Real-Time PCR System (Applied Biosystems, Foster City, CA) using PowerUp SYBR-Green Master Mix kit (Applied Biosystems, Foster City, CA). All samples were assayed in triplicate, and data were normalized to endogenous control *Hprt1*. Relative RNA expression levels were calculated using the ^ΔΔ^Ct method. The primers are listed in Additional file 1: Table S2.

### Western blotting

A total 30 mg left lung tissues was homogenized in 300 μl RIPA buffer (Invitrogen, Carlsbad, CA) supplemented with a protease inhibitor cocktail (Servicebio, Wuhan, China). After determining the protein concentration, the equal amounts of protein were separated on NuPAGE 10% Bis–Tris gels (Invitrogen, Carlsbad, CA) and transferred onto polyvinylidene difluoride (PVDF) membranes. After blocking in 5% nonfat milk at room temperature for 60 min, membranes were incubated with the primary antibodies overnight at 4 °C. The membranes were washed three times for 30 min with TBST (containing 0.1% Tween-20), and then incubated with secondary antibodies. After final washes with TBST, the signals were detected using ECL chemiluminescence reagent Kit (Pierce, Rockford, IL, USA). The primary antibodies performed in this study as showed in Additional file 1: Table S1.

### Histology and immunofluorescence (IF)

The right lung tissues were immediately fixed in 10% neutral buffered formalin for 24 h and go through dehydration, clearing and paraffin embedding. Sections were mounted on positively charged slides after cutting at 4 μm thick, baked at 65 °C for 1 h and then stored at room temperature (RT) for later use. Fibrosis was performed using mason’s trichrome (Genmed Scientifics, Wilmington, DE, USA) and Sirius red stain (Servicebio, Wuhan, China) following the protocols of manufactures. For immunofluorescence (IF) assay, the slides were incubated with xylol and descending concentrations of ethanol. Endogenous peroxidases were blocked by using 0.3% H_2_O_2_ for 10 min at RT. After antigen retrieval, blocking was performed using 5% bovine serum albumin for 30 min at RT. The antibodies used here were listed on Additional file 1: Table S1.

### Hydroxyproline assay

The amount of collagen in the lung tissues was determined by a Hydroxyproline Assay Kit according to the manufacturer's protocol (Cat. No. MAK008-1KT, Sigma-Aldrich, St. Louis, MO). Briefly, about 10 mg lung tissues were homogenized in 100 μl water with 100 μl concentrated hydrochloric acid (HCl, 12 M) and hydrolyzed at 120 °C for 3 h. Transfer 20 μl of supernatant at 60 °C until completely desiccated. Chloramine T/Oxidation Buffer Mixture was added at room temperature for 5 min, followed by the addition of Diluted DMAB Reagent and incubation at 60 °C for 90 min. Measure the absorbance of samples and standards at 560 nm, and hydroxyproline content was expressed as μg per mg lung tissue.

### Statistical analysis

All data were expressed as mean ± SD (standard deviation). For comparisons of different groups, statistical significance was determined by Student's t-test or ANOVA analysis. P-value less than 0.05 was considered statistically significant.

## Results

### *H19* is up-regulated in fibrotic lungs

**Fig. 1:**
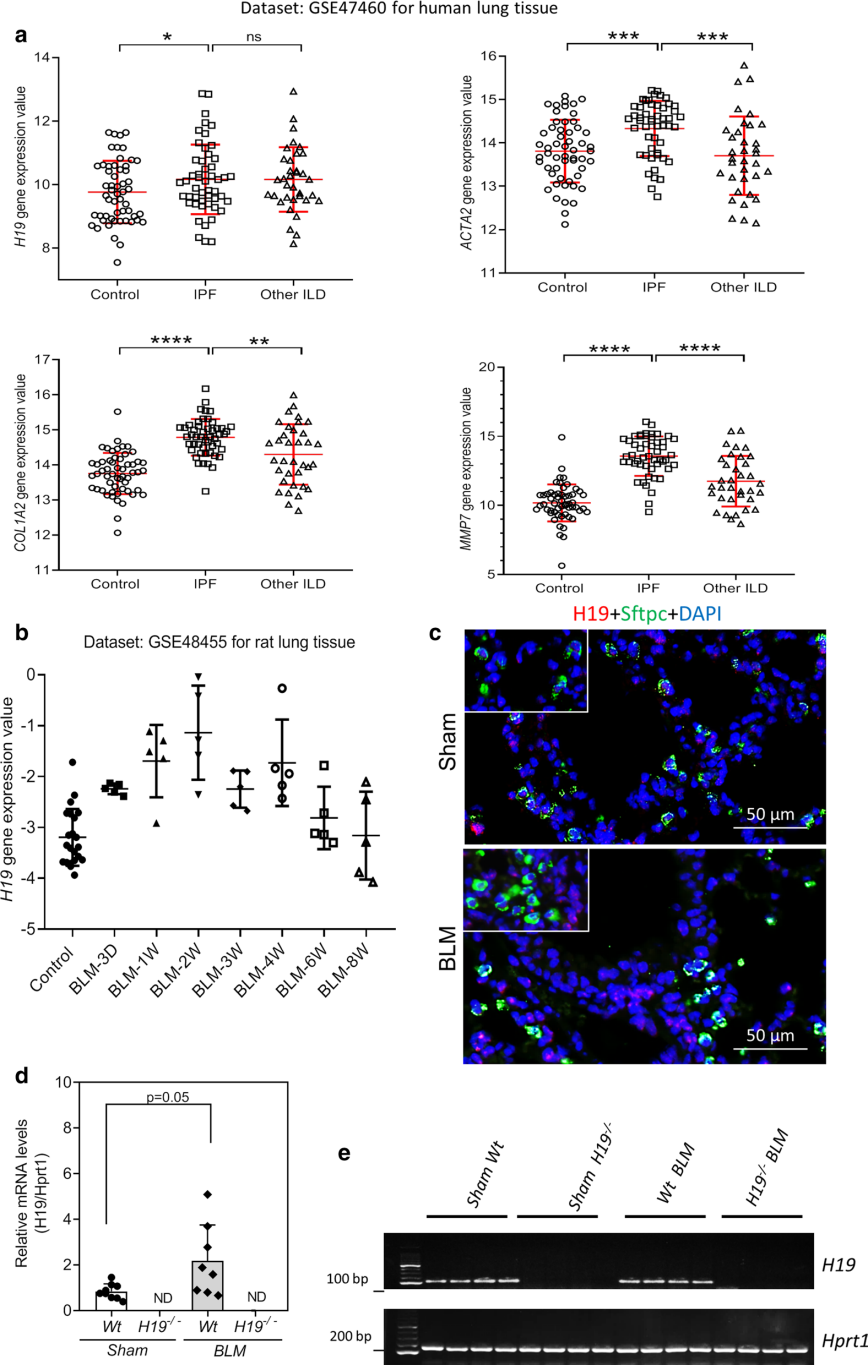
*H19* increased in lungs of pulmonary fibrosis. **a** Data for *H19* mRNA expression extracted from the GEO database, comparing whole extracts of lung tissue from human patients diagnosed with IPF (n = 50) and other interstitial lung diseases (ILD) (n = 35) and from control subjects (n = 55) (GSE47460). **b** Data for *H19* mRNA expression extracted from the GEO microarray database, comparing whole extracts of lung tissue from bleomycin-induced lung fibrosis in rat (n = 5 animals per bleomycin-treated group and n = 22 for saline controls). **c** Representative images of FISH targeting H19 (red) and immunofluorescence (IF) staining of Sftpc in lungs of Sham and bleomycin-treated mice. **d** The relative *H19* mRNA levels in the lungs of Sham wild type (Wt, n = 8), Sham *H19* knockout (*H19*^*−/−*^, n = 6) Wt bleomycin (BLM, n = 8) and *H19*^*−/−*^ BLM (n = 10) mice were determined by real-time RT-PCR. The *Hprt1* was used as an internal control. **e** Representative images of the DNA agarose gels of *H19* and *Hprt1*

Analysis of publicly available datasets showed that lncRNA *H19* expression was more highly expressed in lung tissue from patients with IPF compared to normal lung tissue, but there was no significant difference between IPF patients and other interstitial lung diseases (ILD) (Fig. 1a) [28, 29]. Fibrotic genes, including *ACTA2*, *COL1A2* and *MMP7*, increasingly expressed in lung tissue from patients with IPF compared to lung tissue from patients with other ILD and to normal lung tissue (Fig. 1a) [28, 29]. In addition to, *H19* expression increased upon bleomycin-induced lung fibrosis in rats. *H19* expression peaked after about 2 weeks of bleomycin-treatment, and then *H19* levels returned to an amount comparable to controls (Fig. 1b) [30]. In current study, Fluorescent in situ hybridization (FISH) assay showed that *H19* expression increased in lungs of bleomycin-treated mice (2 weeks) and located at alveolar epithelium and capillaries (Fig. 1c). Furthermore, immunofluorescent staining of surfactant protein C (Sftpc), a marker for type 2 epithelial cells (AEC2s), indicated that *H19* was also expressed and up-regulated in AEC2s following bleomycin-treatment (Fig. 1c). The qRT-PCR assay confirmed that *H19* increasingly expressed in the lungs of mice with bleomycin (BLM) treatment (Fig. 1d, e).

### *H19* deficiency represses bleomycin-induced lung inflammatory response

**Fig. 2:**
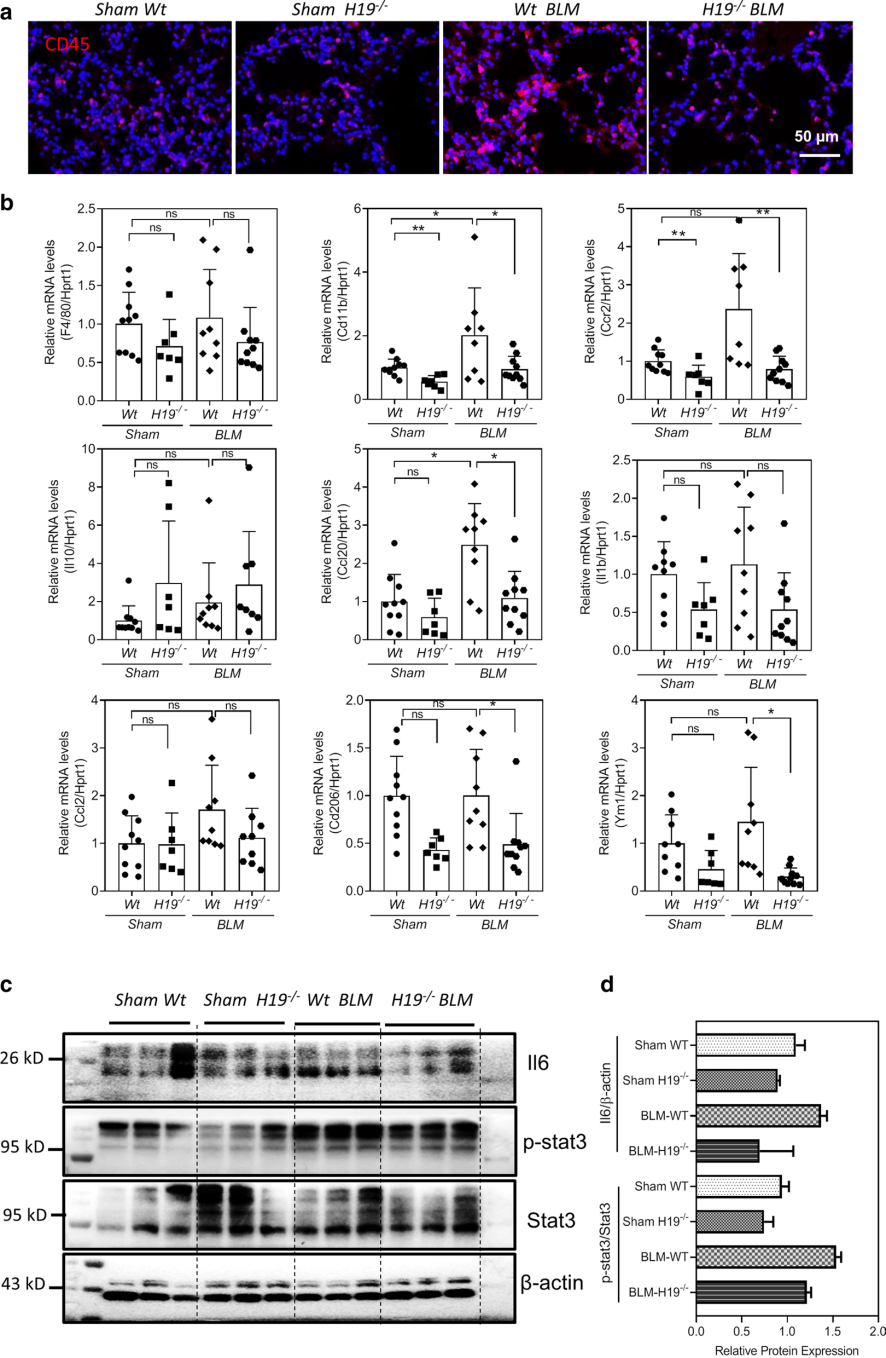
*H19* knockout inhibited bleomycin-induced pulmonary inflammation in mice. **a** Representative immunofluorescence (IF) images of CD45 positive cells in lungs of *Sham Wt* (n = 10), *Sham H19*^*−/−*^ (n = 7), *Wt* BLM (n = 9) and *H19*^*−/−*^ BLM (n = 9) mice. **b** The relative mRNA levels of the inflammatory marker genes including *F4/80*, *CD11b*, *Ccl20*, *IL1b* and *Ym1*, in the lungs of *Sham Wt *(n = 10), *Sham H19*^−/−^ (n = 7), *Wt* BLM (n = 9) and *H19*^*−/−*^ BLM (n = 9) mice. The Hprt1 was used as an internal control. **c** Representative images of the immune blots of Il6, p-Stat3, Stat3 and β-Actin. **d** Relative protein expression levels of Il6 and p-Stat3 were normalized using β-actin or Stat3. Statistical significance: *p < 0.05; **p < 0.01; *ns* not significant

*H19* knock out (*H19*^−/−^) mice here were used to elucidate the roles of *H19* in bleomycin-induced pulmonary inflammation and fibrosis. After 4-week BLM-treatment, immunofluorescence (IF) staining showed the *H19*^*−/−*^ BLM mice had less CD45^+^ cells accumulated in the lungs than that in lungs of *Wt BLM* mice (Fig. 2a). RT-PCR analysis showed that CD11b and Ccr2 genes expression was reduced significantly in lungs of *H19*^*−/−*^ mice when compared to that of *Wt* mice (Fig. 2b). The inflammatory markers, including *F4/80*, *CD11b*, *Ccl20*, *Il1b* and *Ccr2* increased in *Wt BLM* mice compared to *WT* sham mice, but decreased in *H19*^*−/−*^ BLM mice (Fig. 2b). Western blot results indicated that protein expression levels of Il6 and p-Stat3 were decreased in lungs of *H19*^−/−^ BLM mice relative to the *Wt* BLM (Fig. 2c, d).

### *H19* knockout ameliorates bleomycin-induced pulmonary fibrosis

**Fig. 3:**
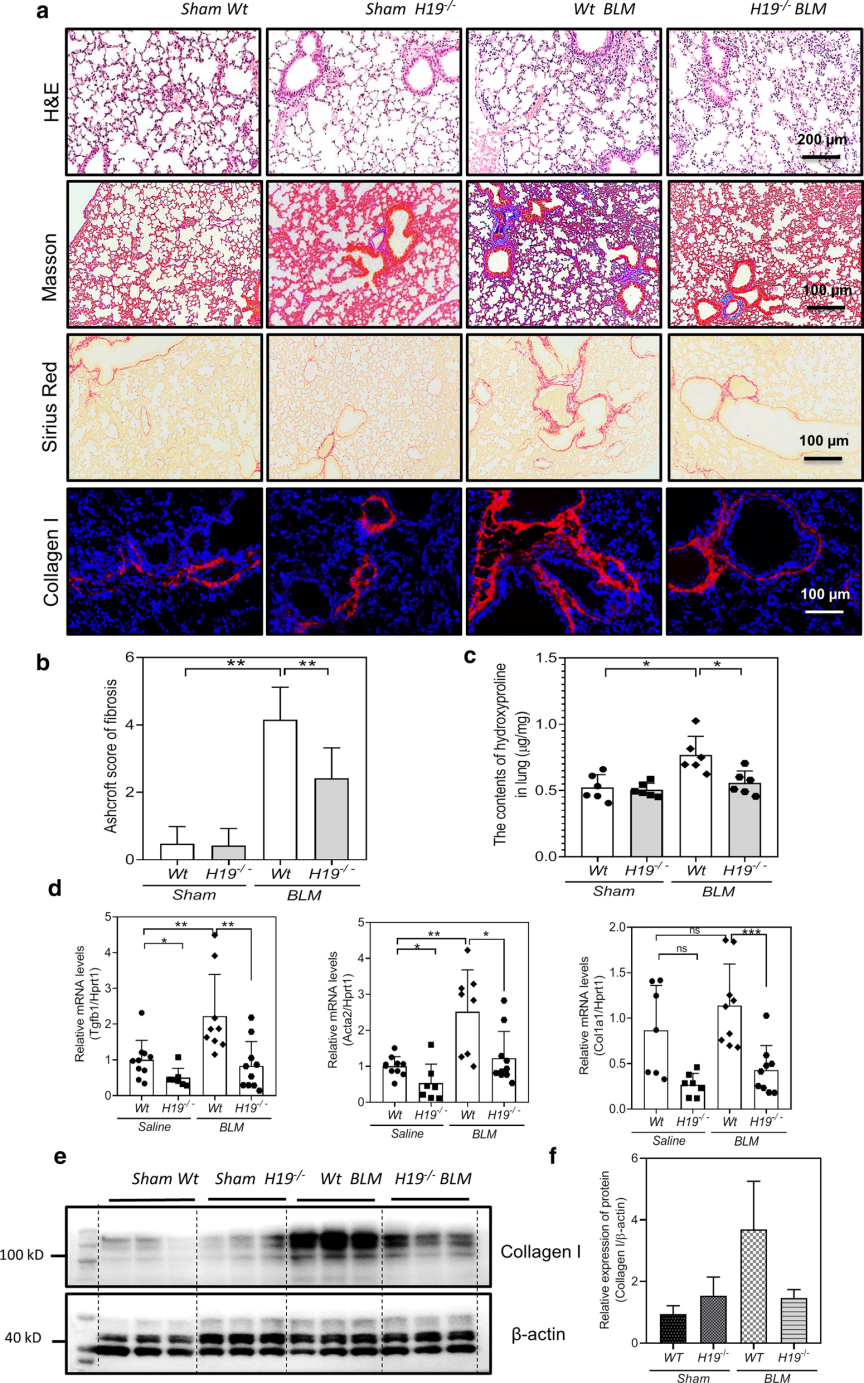
*H19* deficiency ameliorated bleomycin-induced pulmonary fibrosis in mice. **a** Representative images of hematoxylin–eosin (H&E) staining, masson’s trichrome staining, Sirius red staining and collagen I immunofluorescence staining of *Sham Wt*, *Sham H19*^*−/−*^, *Wt* BLM and *H19*^*−/−*^ BLM mice. **b** Ashcroft score for *Sham Wt* (n = 10), Sham *H19*^*−/−*^ (n = 7), *Wt* BLM (n = 9) and *H19*^*−/−*^ BLM (n = 9) mice. **c** Quantifying collagen content with hydroxyproline assay in the lungs of *Sham Wt*, *Sham H19*^*−/−*^, *Wt* BLM and *H19*^*−/−*^ BLM mice. Each group, n = 6–8. **d** Real-time PCR analysis for *Tgfb1*, *Col1a1* and *Acta2* in the lungs of *Sham Wt*, *Sham H19*^*−/−*^, *Wt* BLM and *H19*^*−/−*^ BLM mice. Each group, n = 8–10. **e** Western blot analysis for collagen type I the lungs of *Sham Wt*, *Sham H19*^*−/−*^, *Wt* BLM and *H19*^*−/−*^ BLM mice. **f** Quantification of the panel (**e**)

As shown in Fig. 3, histopathological analysis firstly showed reduced fibrosis in the *H19*^*−/−*^ BLM mice (Fig. 3a, b). The haematoxylin–eosin (H&E) staining and Collagen I immunofluorescence (IF) staining showed the fibrosis increased in *Wt* BLM mice compared to the *Wt* sham mice, but not in lungs of *H19*^*−/−*^ BLM mice (Fig. 3a). The Masson’s Trichrome staining and Sirius red staining further indicated that 4-week BLM significantly induced lung fibrosis in *WT* mice, but had much less impact in *H19*^*−/−*^ mice (Fig. 3a). Consistently, quantitation of lung fibrosis in a blinded manner revealed the Ashcroft score decreased significantly in *H19*^*−/−*^ BLM mice when compared to *Wt* BLM mice (Fig. 3b). Furthermore, the pulmonary hydroxyproline levels were significantly increased in *Wt* BLM mice, but not in *H19*^*−/−*^ BLM mice (Fig. 3c). At molecular level, *H19*^*−/−*^ mice had decreased expression of *Tgfb1* and *Acta2* mRNA in the lungs in relation to *Wt* animals (Fig. 3d). The mRNA levels of *Tgfb1*, *Acta2* and *Col1a1* (Fig. 3d) and protein expression of Col1a1 (Fig. 3e, f) reduced in *H19*^*−/−*^ BLM mice compared to the *Wt* BLM mice.

### *H19* depletion attenuated the pathways of TGF-β/Smad and S1pr1/Sphk2 in fibrotic lungs

**Fig. 4:**
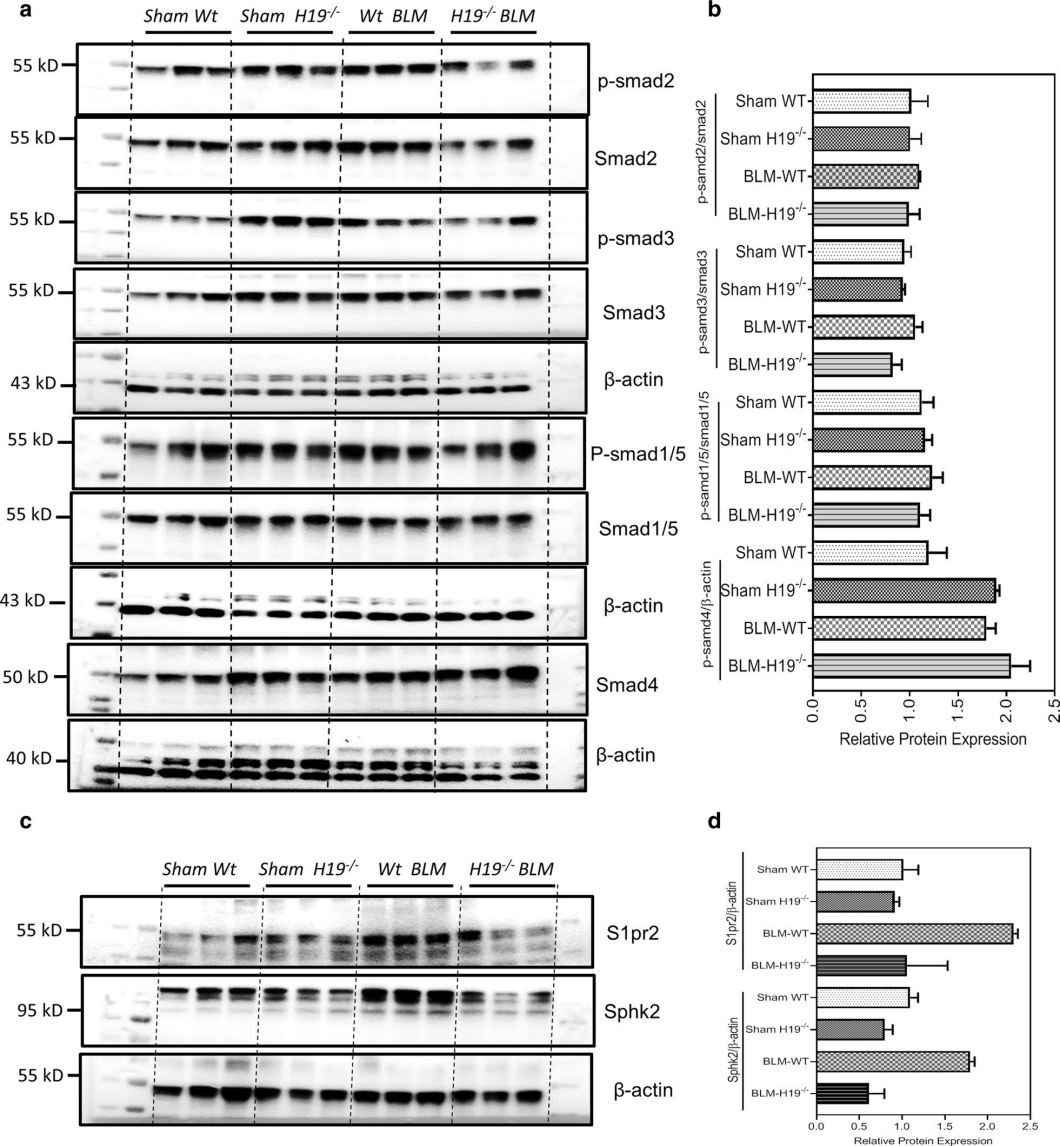
*H19* depletion altered the TGF-β/Smads and S1pr2/Sphk2 pathways in lungs of bleomycin-induced mice. **a** Western blot analysis for Smad1/5, Smad2, Smad3, Samd4 and their phosphorylated forms the lungs of *Sham Wt*, *Sham H19*^*−/−*^, *Wt* BLM and *H19*^*−/−*^ BLM mice. **b** Quantification of the panel (**a**). **c** Western blot analysis for S1pr2 and Sphk2 in the lungs of *Sham Wt*, *Sham H19*^*−/−*^, *Wt* BLM and *H19*^*−/−*^ BLM mice. **d** Quantification of the panel (**c**)

TGF-β/Smad signaling is the key regulating pathway in fibrogenesis [31]. In this study, we showed that the TGF-β mRNA level and protein levels of p-Smad2 and p-Smad3 were increased in the *Wt* BLM mice, compared with *Wt sham* mice, while these proteins reduced in *H19*^*−/−*^ BLM mice (Fig. 4a, b). Our previous study had reported that S1pr2 and SphK2 played an important role in promoting liver fibrosis [20]. As shown in Fig. 4, Western blot results indicated that protein expression levels of S1pr2 and SphK2 were increased in lungs of *Wt* BLM mice compared to *Wt sham* mice, but decreased in lungs of *H19*^*−/−*^ BLM (Fig. 4c, d).

### *H19* deficiency decreased AEC2s proliferation in lungs of bleomycin-treated mice

**Fig. 5:**
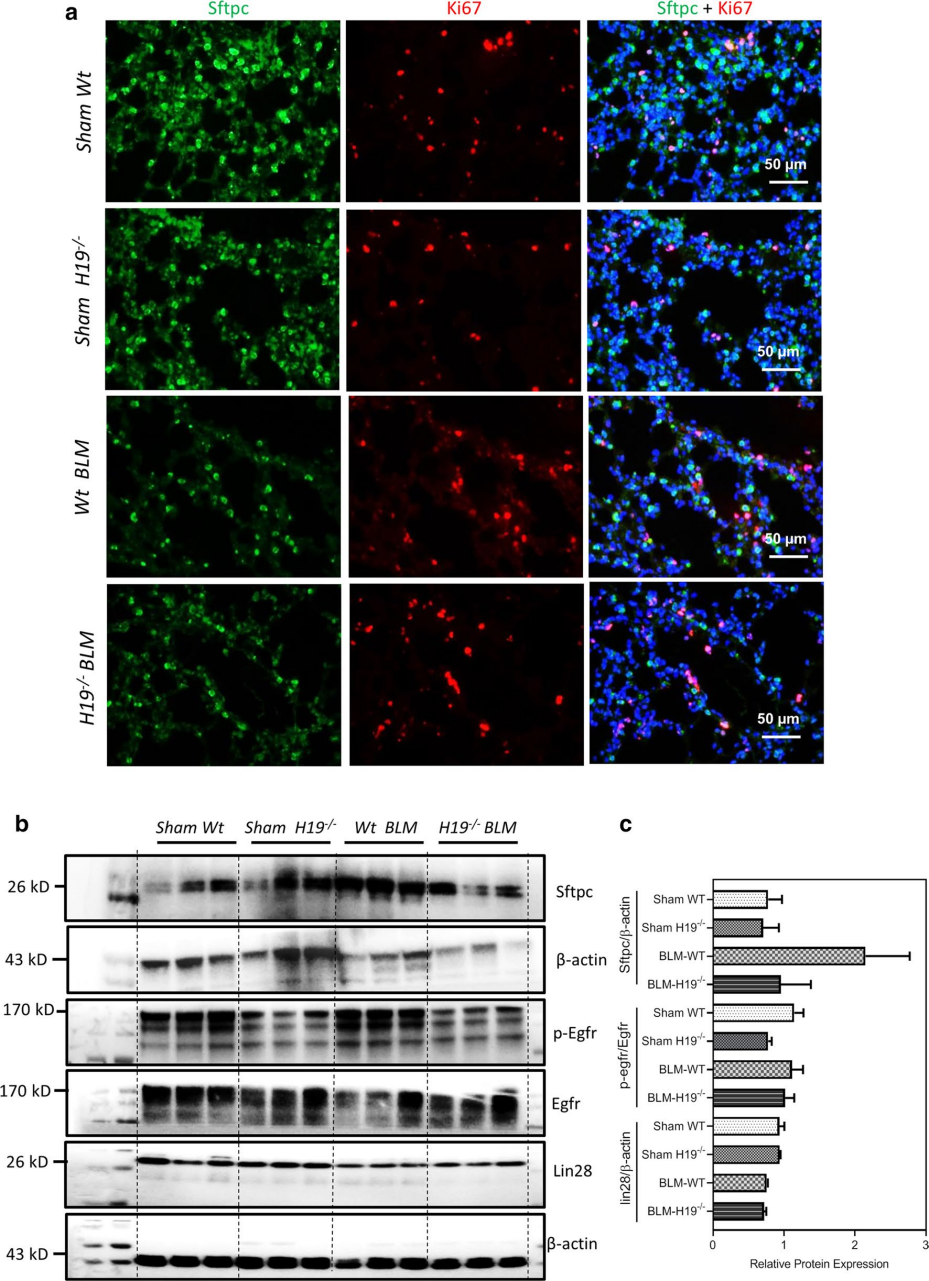
*H19* depletion reduced cell proliferation in lungs of bleomycin-induced mice. **a** Representative images of co-staining for Sftpc and Ki67 in the lungs of *Sham Wt*, *Sham H19*^*−/−*^, *Wt* BLM and *H19*^*−/−*^ BLM mice. **b** Western blot analysis for Sftpc, p-Egfr, Egfr, Lin28 and β-actin in the lungs of *Sham Wt*, *Sham H19*^*−/−*^, *Wt* BLM and *H19*^*−/−*^ BLM mice

In the lung sections from the *H19*^−/−^ BLM mice, immunofluorescence results showed that expression of Sftpc was decreased relative to that of the *Wt* BLM mice (Fig. 5a). Additionally, the number of Ki67-positive cells was also reduced in the *H19*^*−/−*^ BLM mice compared to the *Wt* BLM mice (Fig. 5a and Additional file 1: Figure S1). Consistently, western blot results indicated that protein expression levels of Sftpc reduced in the *H19*^*−/−*^ BLM mice compared to the *Wt* BLM mice (Fig. 5b, c). In addition to, the proteins of p-Egfr decreased in lungs of *H19*^*−/−*^ BLM mice compared to the *Wt* BLM mice (Fig. 5b, c).

## Discussion

Presently, the poor understanding in the pathogenesis of IPF has resulted in a lack of effective therapies. In the current study, we showed that the lncRNA *H19* was up-regulated in the fibrotic lungs of IPF patients and bleomycin-treated mice. Functionally, *H19* deficiency reduced pulmonary inflammation and inhibiting Il6/Stat3 signaling. *H19* knockout ameliorated bleomycin-induced pulmonary fibrosis through attenuating the TGF-β/Smad and S1pr2/Sphk2 pathways. Moreover, we also indicated that *H19* expressed in the type 2 epithelial cells (AEC2s) and contributed to the proliferation of AEC2s.

*H19* is an imprinted and maternally expressed transcript, which is one of the few well-characterized lncRNA [32, 33]. Aberrant expression of *H19* has been related to a variety of human diseases [34–38]. Using a public datasets, it showed that IPF patients had higher levels of *H19* mRNA in lungs when compared to the control subjects. Similarly, *H19* mRNA also increased in a model of bleomycin-induced pulmonary fibrosis. In current study, we also showed that *H19* expression increased in lungs of bleomycin-treated mice and located at alveolar epithelium and capillaries. From the above findings, we hypothesize that *H19* may play an important role in the pathogenesis of IPF. To realize our aim of this study, we firstly generated a *H19* deficiency mouse (*H19*^*−/−*^).

Pulmonary inflammation and fibrosis caused by repetitive lung injury underlies the IPF. In vivo studies, we showed that *H19*^*−/−*^ mice could attenuate bleomycin-induced pulmonary inflammation. During the bleomycin-induced mice, the CD11b and Ccr2 mRNA expression increased the lung of wild type mice, but not in that of *H19*^*−/−*^ mice. Recently, Li et al., reported that *H19* significantly induced the expression and secretion of chemokine (C–C motif) ligand 2 (CCL-2) that could accumulate the monocytes from circulation into livers [39]. It thus suggests that *H19* contributes to the pulmonary inflammation may via attracting the CD11b monocytes into the lung after injures. The signaling studies presented here revealed that Il6/Stat3 was reduced in the bleomycin damaged lungs of *H19*^*−/−*^ mice. In the injured lung, STAT3 rapidly activated and increased the production of the proinflammatory molecules *IL1β*, *IL6*, *TNF-α*, *iNOS* and *CCL2* [40–43]. It thus propose that *H19*^*−/−*^ mice reduced bleomycin-induced pulmonary inflammation may through attenuating the Il6/Stat3 signaling. In vivo studies further revealed *H19* deficiency significantly reduced bleomycin-induced pulmonary fibrosis. TGF-β/smad signaling is one of the key pathways responsible for pulmonary fibrosis [31, 44–46]. The current study indicated that *H19*^−/−^ mice attenuated the TGF-β/smad signaling in bleomycin damaged lungs by reducing the expression of *Tgfb1* mRNA and activated the Smad2/3 protein. Consistently, in vitro study revealed that *H19* can target miR-140 and regulate the TGF-β/Smad3 pathway [24]. Moreover, *H19* could enhance TGF-β signaling in both hepatic stellate cells and hepatocytes and facilitate liver fibrosis [47]. *H19* has been reported to accelerate TGF-β1-induced tenogenic differentiation in vitro and promoted tendon healing in a mouse tendon defect model [48]. Sphingosine-1-phosphate and its receptor S1pr2 have been shown to promote lung fibrosis [49–53]. Our previous study also showed that *H19* could activate the S1pr2/SphK2 signalling pathway in the cholestatic livers. The current study indicated that S1pr2/SphK2 signalling was activated in the bleomycin-treated lungs, but these effects were attenuated by *H19* knockout. We thus propose that *H19* contribute to lung fibrosis of IPF may via regulating both TGF-β/smad and S1pr2/SphK2 signalling. Epidermal growth factor receptor (EGFR) is a major driver of lung adenocarcinoma, which is essential to lung cancer cell proliferation [54]. The present study also showed *H19* deficiency repressed EGFR activating and suppressed the ACE2s proliferation.

## Conclusion

In summary, we demonstrated that the *H19* is a potential therapeutic target for IPF patients. We propose two novel mechanisms underlying *H19* activity in the pathogenesis of IPF. *H19* knockout inhibits pulmonary inflammation by attenuating the Il6/Stat3 signaling. *H19* acts as a profibrotic lncRNA in the lung of IPF via regulating the TGFβ/Smad and S1pr2/Sphk2.

## Supplementary information

**Additional file 1: Table S1.** Antibody information. **Table S2.** Sequences of primers. **Figure S1.** Quantification of the Fig. 5a.

## Data Availability

Original data can be requested from corresponding author.

## Abbreviations

IPF: Idiopathic pulmonary fibrosis
lncRNA: Long non-coding RNA *H19*
UIP: Usual interstitial pneumonia
RT-PCR: Real-time polymerase chain reaction
IF: Immunofluorescence
H&E: Hematoxylin–eosin
EGFR: Epidermal growth factor
TGF-β receptor: Transforming growth factor-β
S1PR2: Sphingosine-1-phosphate receptor 2
SphK2: Sphingosine kinase 2
ACTA2: Alpha 2 smooth muscle actin
COL1A2: Collagen type I alpha 2
MMP7: Matrix metalloproteinase 7
