# Supplementary material for: Long non-coding RNA H19 deficiency ameliorates bleomycin-induced pulmonary inflammation and fibrosis
Source: Respir Res. 2020 Nov 2;21:290. doi: 10.1186/s12931-020-01534-6 (PMC7607673; doi:10.1186/s12931-020-01534-6)
Supplement: Supplementary file 1 — Additional file 1: Table S1. Antibody information. Table S2. Sequences of primers. Figure S1. Quantification of the Fig. 5a. [file 12931_2020_1534_MOESM1_ESM.docx]

**Table S1 Antibody information**

| **Antibody** | **Source** | **Catalog#** | **Application/dilution** |
| --- | --- | --- | --- |
| beta-tubulin | Bioss Inc | bs-33034M | WB(1:2000) |
| beta-actin | Cell Signaling Technology | 4970S | WB(1:1500) |
| Collagen I | Abcam | ab138492 | WB(1:1000),IHC/IF(1:200) |
| P-STAT3 | Cell Signaling Technology | 9145S | WB(1:1000) |
| STAT3 | Cell Signaling Technology | 9139P | WB(1:1000) |
| IL6 | Abcam | ab6672 | WB(1:500) |
| Sphk2 | Proteintech | 17096-I-AP | WB(1:1000) |
| S1pr2 | Proteintech | 21180-I-AP | WB(1:1000) |
| P-smad2 | Cell Signaling Technology | 3108P | WB(1:1000) |
| P-smad3 | Cell Signaling Technology | 9520P | WB(1:1000) |
| P-smad1/5 | Cell Signaling Technology | 9516P | WB(1:1000) |
| smad1 | Cell Signaling Technology | 6944P | WB(1:1000) |
| smad4 | Cell Signaling Technology | 9515P | WB(1:1000) |
| lin28 | Bioss Inc | bs-8443R | WB(1:1000) |
| P-EFGR | Cell Signaling Technology | 2236L | WB(1:1000) |
| EGFR | Cell Signaling Technology | 2232L | WB(1:1000) |
| SFTPC | invitrogen | PA5-71680 | WB(1:1000),IF (1:200) |
| CD45 | Servicebio | GB11066 | IF(1:200) |
| KI67 | Servicebio | GB13030-2 | IF(1:100) |
| Collagen I | Servicebio | GB11022-3 | IF(1:200) |

**Table S2 Sequences of primers**

| Hprt1 | Forward (5'-3') | AAGCTTGCTGGTGAAAAGGA |
| --- | --- | --- |
|  | Reverse (5'-3') | TTGCGCTCATCTTAGGCTTT |
| H19 | Forward (5'-3') | GGGTCTGTTTCTTTACTT |
|  | Reverse (5'-3') | TAGCACCATTTCTTTCAT |
| CD11b | Forward (5'-3') | AGCTTGGCTTTTTCAAGCGG |
|  | Reverse (5'-3') | AAAGGCCGTTACTGAGGTGG |
| Ccl2 | Forward (5'-3') | CAGGTCCCTGTCATGCTTCT |
|  | Reverse (5'-3') | GAGTGGGGCGTTAACTGCAT |
| Ccl20 | Forward (5'-3') | ATGGCCGATGAAGCTTGTGA |
|  | Reverse (5'-3') | CTCCTTGGGCTGTGTCCAAT |
| CD206 | Forward (5'-3') | GTGGAGTGATGGAACCCCAG |
|  | Reverse (5'-3') | CTGTCCGCCCAGTATCCATC |
| Tgfb1 | Forward (5'-3') | AGGGCTACCATGCCAACTTC |
|  | Reverse (5'-3') | CCACGTAGTAGACGATGGGC |
| Col1a1 | Forward (5'-3') | CGACCTCAAGATGTGCCACT |
|  | Reverse (5'-3') | CCATCGGTCATGCTCTCTCC |
| Acta2 | Forward (5'-3') | CCGACCGAATGCAGAAGGA |
|  | Reverse (5'-3') | ACAGAGTATTTGCGCTCCGAA |
| F4/80 | Forward (5'-3') | TGTCTGAAGATTCTCAAAACATGGA |
|  | Reverse (5'-3') | TGGAACACCACAAGAAAGTGC |
| Ccr2 | Forward (5'-3') | GCCATCATAAAGGAGCCATACC |
|  | Reverse (5'-3') | ATGCCGTGGATGAACTGAGG |
| Il10 | Reverse (5'-3') | TAAGGCTGGCCACACTTGAG |
|  | Forward (5'-3') | GTTTTCAGGGATGAAGCGGC |
| Ym1 | Forward (5'-3') | GGGCCCTTATTGAGAGGAGC |
|  | Reverse (5'-3') | CCAGCTGGTACAGCAGACAA |

**Figure S1 Quantification of the figure 5A**
